# Large-scale gene analysis of rabbit atherosclerosis to discover new biomarkers for coronary artery disease

**DOI:** 10.1098/rsob.180238

**Published:** 2019-01-23

**Authors:** Xiaolan Yu, Wen Guan, Yang Zhang, Qing Deng, Jingjing Li, Hao Ye, Shaorong Deng, Wei Han, Yan Yu

**Affiliations:** 1 Shanghai Municipality Key Laboratory of Veterinary Biotechnology, School of Agriculture and Biology, Shanghai Jiao Tong University, Shanghai, People's Republic of China; 2 Laboratory of Regeneromics, School of Pharmacy, Shanghai Jiao Tong University, Shanghai, People's Republic of China; 3 Department of Biochemistry, Zhongshan School of Medicine, Sun Yat-sen University, GuangZhou, People's Republic of China

**Keywords:** gene chip, atherosclerosis, regression, biomarkers, coronary artery disease, New Zealand white rabbits

## Abstract

Atherosclerosis is the pathological basis of coronary artery disease (CAD) and causes high mortality. Thus, early detection is thought to be crucial in reducing the risk of CAD. Uncovering the mechanisms of the progression and regression of atherosclerosis will provide insights into discovering novel biomarkers to identify subjects at risk for CAD and improve prevention. We established atherosclerosis progression and regression in a rabbit model. Then, we extracted mRNA of the abdominal aorta from control, model and recovery groups to perform gene chip analysis. Candidate biomarkers were screened by large-scale gene analysis and validated in patients with CAD or with CAD recovery by ELISA. The differentially expressed genes in the progression and regression of atherosclerosis were mainly enriched in four clusters. Genes associated with inflammation and extracellular matrix were returned to normal or close-to-normal levels much earlier than genes associated with metabolism and sarcoplasmic proliferation, and they were maintained downregulated or upregulated after feeding a normal diet. We then selected four candidate biomarkers and found that lipoprotein lipase (LPL), bone morphogenetic protein 7 and somatostatin concentrations could indicate CAD diagnosis. In addition, LPL and macrophage cationic peptide 2 can be indicators of the prognosis of CAD. Molecular changes during the progression and regression of atherosclerosis in rabbits were revealed, and candidate regulators were identified. The identified factors could be used as novel biomarkers and targets for improving the diagnosis and prognosis of human CAD in the future.

## 1. Introduction

Atherosclerosis is the pathological basis of coronary artery disease (CAD), threatening human health and leading to high mortality. Despite percutaneous coronary intervention (PCI) being a well established and useful technique for CAD, various other factors such as inflammation and insulin resistance can lead to restenosis and threaten survival [1,2]. Thus, early detection and control of risk factors is thought to be crucial in reducing the risk of CAD. Uncovering additional mechanisms of the progression and regression of atherosclerosis will provide insights into discovering novel biomarkers to identify subjects at risk for CAD and enable the administration of interventional approaches.

Establishing an appropriate experimental animal model is essential for studying the pathophysiology of atherosclerosis. Animal models should reflect the most important aspects of lipid metabolism and the features of lesions, such as fatty streaks, fibrous plaques and complicated plaques with calcification [3], that are observed in human patients. However, there is no single animal model that fulfils all these requirements. Therefore, illustrating the diverse aspects of atherosclerosis requires different animals [4]. Rabbits were the first and remain one of the best models for studying lipoprotein metabolism and atherosclerosis. Considering the convenience of measuring atherosclerotic plaques by intravascular ultrasound, we preferred to use rabbits as model animals. In addition, regression of atherosclerosis has been demonstrated in cholesterol-fed rabbits after withdrawal of cholesterol from the diet [5,6]. Furthermore, the addition of certain substances, such as high-density lipoprotein cholesterol (HDL-c), paclitaxel or phosphatidylcholine, into the normal diet accelerated the regression of atherosclerosis [7–9]. In our previous research, we found that the regression of atherosclerosis by a normal diet requires eight weeks or more. Some studies have shown that caloric restriction is the most effective and reproducible intervention against obesity, diabetes and atherosclerosis through increasing the HDL-c levels and decreasing the levels of total cholesterol (TCH) and low-density lipoprotein cholesterol (LDL-c) [10–12]. To speed the regression of atherosclerosis, we designed an atherosclerosis rapid regression experiment using a caloric restriction diet instead of a normal diet.

Atherosclerosis is a dynamic process with intercalating periods of quiescence and activity during which plaque growth occurs [13]. Novel biomarkers may contribute to our understanding of the natural history of atherosclerotic plaques and may help to identify periods of disease activity related to the prediction and prognosis of coronary events. Large-scale gene expression analyses have been performed to reveal the molecular changes of atherosclerosis at the mRNA level [14,15]. Most of these studies have focused on the formation of atherosclerosis rather than the regression of atherosclerosis. In our study, we applied gene chip analysis of the abdominal aorta to simultaneously delineate the molecular characteristics of atherosclerosis progression and regression, to identify important genes related to the diagnosis and prognosis of atherosclerosis, and to discover new biomarkers for human CAD.

## 2. Methods

### 2.1. Animal and study design

Twelve adult (3 months old, 3.0 kg), male, New Zealand white rabbits were purchased from SLACCAS (Shanghai, China). Eight randomly selected rabbits were fed a high-cholesterol diet (purified rabbit chow supplemented with 1% cholesterol and 6% peanut oil) (SLACCAS, Shanghai, China) for four weeks. After receiving this diet for three days, balloon injury of the abdominal aorta was performed. Under general anaesthesia (acepromazine (0.75 mg kg^−1^), xylazine (2.5 mg kg^−1^) and ketamine (35 mg kg^−1^)), balloon injury of the abdominal aortic wall was performed using a 4F Fogarty catheter introduced through a right femoral artery cutdown. When the catheter was advanced to the diaphragm, the balloon was inflated and the catheter was gently retracted towards the iliofemoral artery. This procedure was repeated three times [16]. Four randomly selected rabbits were killed at four weeks after plaque formation (model group or AS30). The remaining four animals were switched to a caloric restriction diet (chow with no added cholesterol or fat, reduction in daily intake by 20 per cent of ad libitum consumption) for four more weeks to monitor regression of atherosclerosis (recovery group or AS60). The other four uninjured rabbits were fed a normal diet (chow with no added cholesterol or fat, ad libitum feeding) and used as a control (control group or AS0) for the model groups.

**Figure 1.**
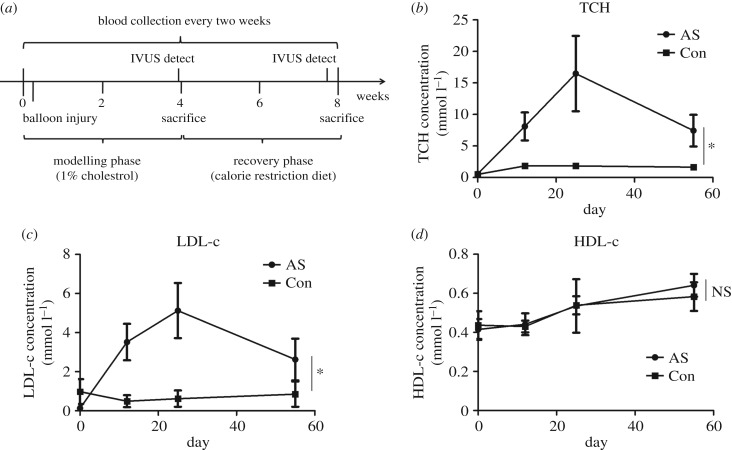
Lipid profiles of rabbit atherosclerosis. (*a*) Experimental timeline. Rabbits were fed with a high-cholesterol diet (purified rabbit chow supplemented with 1% cholesterol) for four weeks. Three days into the diet, balloon injury of the abdominal aorta was performed. After four weeks of high cholesterol diet, rabbits switch to the calorie restriction diet for four more weeks (chow with no added cholesterol or fat, reduction of daily intake by 20% of ad libitum feeding). Blood was collected at 0, 2, 4 and 8 weeks for lipid measurement. Intravascular ultrasound imaging was performed at the end of the fourth and eighth weeks. (*b*–*d*) TCH, LDL-c and HDL-c concentrations during the progression and regression of atherosclerosis. Con, control (*n* = 4); AS, atherosclerosis (AS30, *n* = 8; AS60, *n* = 4). Data were reported as means ± s.d. and between-group comparisons were performed by Students' *t*-test. A *p*-value of less than 0.05 was considered significant.

Blood was collected at each point at 0, 2, 4 and 8 weeks for lipid measurement. At the end of the fourth and eighth weeks, we used intravascular ultrasound (IVUS) imaging to detect characteristic lesions in the arterial wall (figure 1*a*).

Animal experiments were performed under authorization of the Animal Care and Use Committee of Shanghai Jiao Tong University.

### 2.2. Intravascular ultrasound imaging and analysis

IVUS imaging was performed according to the previous study [17]. In brief, a rotating 40 MHz transducer, which consisted of a motorized transducer pullback system and a commercial scanner (Galaxy, Boston Scientific, Natick, MA, USA), was operated with a 2.9 Fr sheath-based catheter (Atlantis SR Pro, Boston Scientific, Natick, MA, USA). The IVUS catheter was advanced into the diaphragm, and imaging (an automated transducer pullback at 0.5 mm s^−1^) was performed back to the iliofemoral artery after intra-coronary administration of 200 mg of nitroglycerin. The images were stored in the computer system (TomTec Imaging Systems, Munich, Germany) and then recorded on digital media (CD/DVD) for off-line quantitative analysis using commercially available analysis software (INDEC Systems, Santa Clara, CA, USA). Vessel area, lumen area and mean neointimal area were measured.

### 2.3. Blood sampling and lipid measurement

In the morning after 12 h of fasting, 5 ml blood samples were collected from the ear vein into pro-coagulant tubes. The serum was then collected after centrifuging at 3000*g* for 15 min at 4°C and stored at −80°C until use. TCH, HDL-c and LDL-c were measured according to the protocols of the commercial kits (TCH: A111; HDL-c: A112; LDL-c: A113; Nanjing Jiancheng Biological Institute, Nanjing, China).

### 2.4. Histology analysis

At the indicated time points (4 or 8 weeks), after IVUS analysis, rabbits were sacrificed, and the abdominal aortas from the diaphragm to the iliofemoral artery were excised. The aortas were washed in phosphate buffered saline (PBS) and immediately divided into two parts. One section was fixed in RNA Later and stored at −80°C for mRNA extraction. The other section was used for histology analysis; this section was embedded in optimal cutting temperature compound (Sakura Finetek, Northbrook, USA) for cryosectioning under liquid nitrogen and stored at −80°C until processing or fixed in PBS buffered 10% formalin for paraffin sectioning. Ten-micrometre-thick sections were stained with haematoxylin–eosin (HE) or Oil red O for lipids and counterstained with haematoxylin. In addition, the formalin-fixed aortas were stained by Oil red O solution for image acquisition.

An experienced pathologist who was blinded to the groups assessed all histological samples. Intimal thickness was measured as the distance from the inner surface of each arterial wall to the luminal border. Medial thickness was measured as the distance between the internal and external elastic lamina [18]. In the analysis of Oil-red-O- or HE-stained abdominal aortas, the area was calculated using ImageJ in three random 100× microscopic fields.

### 2.5. Sample collection and mRNA preparation

Total RNA was extracted using TRIzol reagent following the manufacturer's instructions and checked for an RNA integrity number (RIN) to inspect RNA integrity by using an Agilent Bioanalyzer 2100 (Agilent Technologies, Santa Clara, CA, USA). Qualified total RNA (RIN ≥ 7) was further purified using an RNeasy micro kit (QIAGEN, GmBH, Germany) and an RNase-Free DNase Set (QIAGEN, GmBH, Germany). One aorta sample was selected from the control, model and recovery groups under the guidance of the pathological sections and IVUS.

### 2.6. Microarray expression analysis

To study the relative gene expression profiles in atherosclerosis, we analysed the relative abundance of mRNA using microarray analysis with the Agilent Array platform (Agilent Technologies, Santa Clara, CA, USA). Sample preparation and microarray hybridization were performed according to the manufacturer's protocols. Briefly, 1 µg of total RNA from each sample was amplified and transcribed into fluorescent cRNA using Agilent's Quick Amp Labelling protocol. The labelled cRNAs were hybridized onto the Rabbit Genome genome-wide 4 × 44k chip (Agilent Technologies, design ID: 020908). After washing the slides, the arrays were scanned using an Agilent scanner (G2565CA). Images were analysed by the Agilent Feature Extraction image analysis software, and image signals were converted into digital signals. Signal values were recorded and marked with ‘P' for present, ‘M' for marginal or ‘A' for absent according to expression level. Quantile normalization and subsequent data processing were performed using the GeneSpring software package (Agilent Technologies). Genes with expression levels that varied at least twofold between any two time points were considered differentially expressed genes, excluding the appearance of two ‘A' values. The selected differentially expressed genes were subjected to hierarchical clustering analysis using Cluster 3.0 (Stanford University, Sandford, CA, USA) and TreeView (University of California, Oakland, CA, USA) tools. Annotation of gene function and fold enrichment analysis was conducted by the DAVID (Databases for Annotation, Visualization and Integrated Discovery) online tools (https://david.ncifcrf.gov/) according to the available instructions. The modified Fisher exact *p*-value for gene-enrichment analysis was calculated, and a *p*-value of less than 0.05 was considered significant.

### 2.7. Quantitative real-time PCR

Real-time PCR reactions were performed with the ABI detection system (Applied Biosystems, Foster City, CA, USA). Reverse transcription was performed using 1 µg of total RNA from each sample and the MMLV Reverse Transcriptase cDNA Synthesis Kit (TaKaRa, Dalian, China), according to the manufacturer's instructions. RT-PCR was performed using SYBR Green (TaKaRa, Dalian, China) in the ABI 7500 Sequence Detection System. PCR primer sequences were designed for the candidate genes and the GAPDH housekeeping gene using Primer Premier 5.0 software, and their sequences are listed in table 1. Each RT-PCR reaction was performed in triplicate. For analysis, fluorescence signal values were obtained using ABI 7500 Sequence Detection System software, and the Ct value of each sample and gene was obtained. The 2^−ΔΔt^ method was used to calculate the differences in gene expression in multiples.

**Table 1.** Primer information for each gene.

GenBank accession number	gene symbol	primer sequences (5′ → 3′)	
NM_001082253.1	GAPDH	sense	GGAGAAAGCTGCTAA
		antisense	ACGACCTGGTCCTCGGTGTA
XM_002724032.2	BMP7	sense	GGGCTTCTCCTACCCCTACA
		antisense	TTGTCGTGTTCCACGAGGTT
NM_001177330.1	LPL	sense	GAAACTCAAGTGGAACAGCGAC
		antisense	TCAGAGACTTGTCGTGGCATTT
NM_001171013.1	MCP2	sense	AAGTCGTAGACCAGCAGCCC
		antisense	GCAGCAGAGTGGGTGGATTCT
XM_002716499.3	SST	sense	CCCAACCAGACGGAGAATGA
		antisense	AGGGATTCTGGGGGATTAGG

### 2.8. Human serum sampling and ELISA

To further study the relationship between candidate genes and the progression and regression of atherosclerosis in humans, we collected serum samples from 16 healthy male human subjects, 28 male patients with CAD and 20 male CAD patients after PCI at Ruijin Hospital (Shanghai, China) between 1 January 2014 and 31 December 2015, respectively. Patients who underwent coronary angiography with coronary lesions exhibiting greater than or equal to 50% narrowing in more than one of the main coronary arteries were diagnosed with CAD. In addition, the CAD patients after PCI without in-stent restenosis in one to two years were categorized as patients with CAD recovery.

Blood samples (5 ml) were taken in the morning after 12 h of fasting and collected into pro-coagulant tubes. Serum was separated within 1 h, and the samples for candidate protein concentration measurements were frozen at −80°C. The remaining serum samples were used for serum lipoprotein analysis. TCH, total triglyceride (TG), LDL-c and HDL-c were measured enzymatically on a Hitachi 912 analyser (Roche Diagnostics, Mannheim, Germany). Plasma glucose concentrations were measured by the glucose oxidase method. Body mass index was calculated as weight (kg) divided by the square of height (m^2^).

We measured the protein concentrations of the candidate genes in serum using ELISA kits according to the manufacturer's standard methods (LPL, IBL, NO 27184; BMP7, R&D, DY354; MCP2, RayBiotech, ELH-CCL8-1; SST, RayBiotech, ELH-Som-1).

All subjects provided written informed consent, and the study protocol was approved by the local ethics committee.

### 2.9. Statistics

All statistical analyses were performed using SPSS18.0 statistical software. Data were reported as the median ± interquartile (IQR), the mean ± s.d., or proportions (%). Comparisons were performed by one-way ANOVA among the three groups and by Student's *t*-test between two groups. The progression of atherosclerosis was compared between the model group (AS30) and the control group (AS0), and the regression of atherosclerosis was compared between the recovery group (AS60) and the model group (AS30). A *p*-value of less than 0.05 was considered significant.

## 3. Results

### 3.1. Lipid profiles of rabbit atherosclerosis

To investigate the gene expression pattern in the progression and regression of atherosclerosis, we established a rabbit model of atherosclerosis by feeding rabbits a high-cholesterol diet and including balloon injury, followed by feeding a caloric restriction diet (figure 1*a*). From the lipid profiles, we found that both serum TCH and LDL-c concentrations were significantly increased during the high-cholesterol diet period (AS30) compared with the control (*p* < 0.05). After reversion to the caloric restriction diet for four weeks (AS60), both serum TCH and LDL-c concentrations were significantly reduced compared with the model group (AS30; *p* < 0.05) (figure 1*b*,*c*). By contrast, the concentrations of HDL-c between the two groups differed without significance at different time points (figure 1*d*).

### 3.2. Intravascular ultrasound of rabbit atherosclerosis

**Figure 2.**
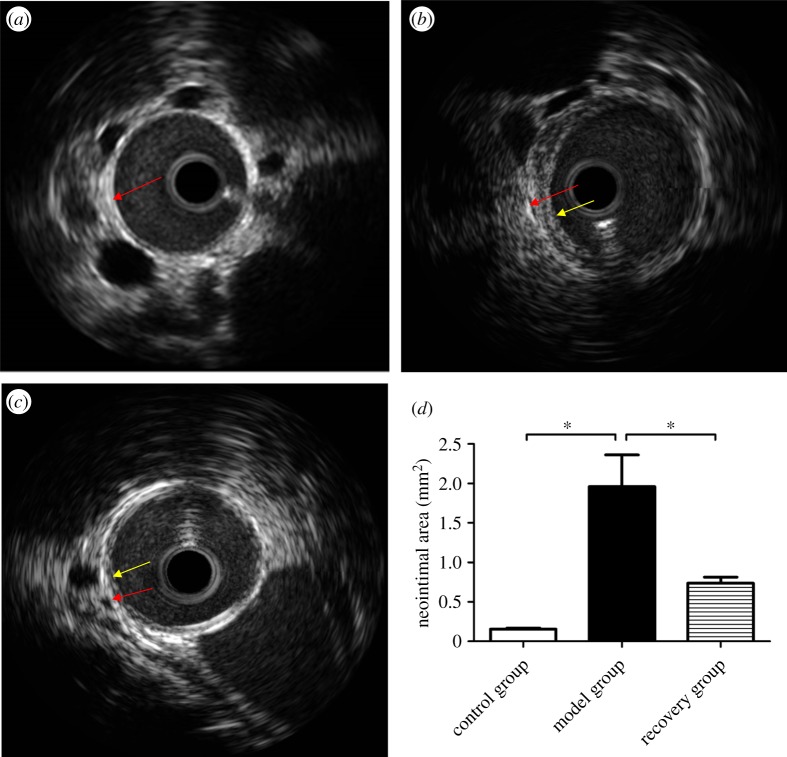
Intravascular ultrasound of rabbit atherosclerosis. Intravascular ultrasound images show representative pictures of rabbit abdominal aorta in control, model and recovery groups, respectively (*a*, *b* and *c*). Obvious neointima was observed in the model group and little neointima was observed in the recovery group, whereas no neointima was observed in the control group. The mean neointimal area was measured and analysed (*d*). The red and yellow arrows indicate the intima and the neointima, respectively. Data were reported as means ± s.d. and among three-group comparisons were performed by ANOVA, *n* = 4. *p < 0.05. A *p*-value of less than 0.05 was considered significant.

We directly detected plaque formation and regression of the rabbit abdominal aorta using IVUS, and the results are shown in figure 2. The vessel intima in the control group was smooth (figure 2*a*); however, there was obvious plaque formation in the model group (figure 2*b*). After reversion to the caloric restriction diet for four weeks, the plaques were significantly reduced (figure 2*c*). The neointimal area was significantly increased in the model group, whereas it was significantly reduced in the recovery group when compared with the model group (control group: 0 mm^2^; model group: 1.96 ± 0.69 mm^2^; recovery group: 0.74 ± 0.14 mm^2^, *p* < 0.05, figure 2*d*).

### 3.3. Histological analysis of rabbit atherosclerosis

**Figure 3.**
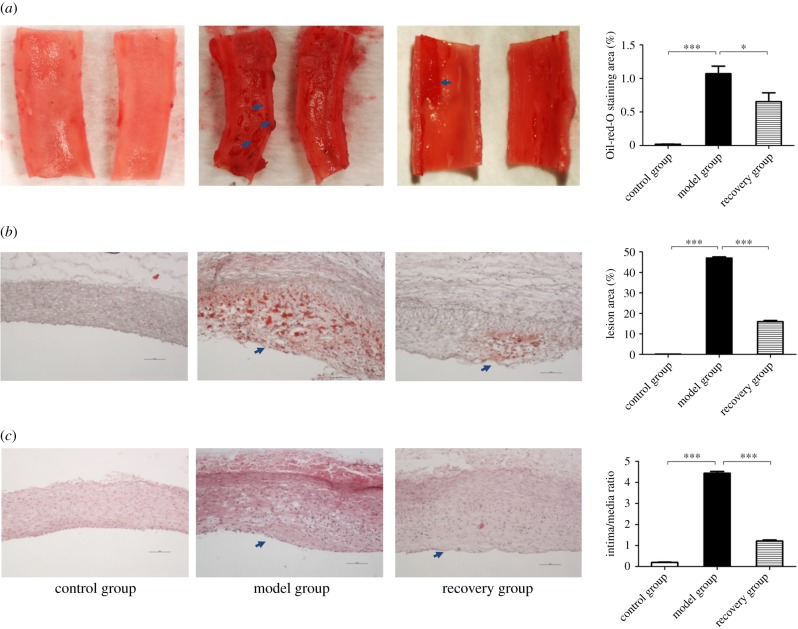
The histology analysis of rabbit atherosclerosis. The whole abdominal aorta was stained using Oil red O solution for acquisition of the overall plaque formation (arrows). The percentage of lesion area was significantly increased in the model group and relatively decreased in the recovery group (*a*). The Oil red O staining of cryosections showed that there were obvious lipid droplets (arrow) in the vascular endothelial cells of the model group while significantly reduced in those of the recovery group (*b*). The HE staining of paraffin sections demonstrated the ratio of intima to media thickness among the three groups. The endothelial cells were significantly proliferated (arrow) in the model group and relatively decreased in the recovery group (*c*). The values of the percentage of lesion area and lipid droplet and the ratio of intima to media thickness were measured by ImageJ software, respectively (displayed on right side). Data were reported as means ± s.d. and among three-group comparisons were performed by ANOVA, *n* = 4. *p < 0.05, ***p < 0.001. A *p*-value of less than 0.05 was considered significant.

To further test whether our atherosclerosis progression and regression model was established successfully, we performed Oil red O and HE staining on the rabbit abdominal aorta. From figure 3*a*, we found that the percentage of the lesion area was significantly increased in the model group and significantly reduced in the recovery group (control group: 0%; model group: 47 ± 0.58%; recovery group: 16 ± 0.57%, *p* < 0.05). We also observed obvious lipid droplets in the vascular endothelial cells of the model group, but they were significantly reduced in the recovery group (control group: 0%; model group: 1.07 ± 0.19%; recovery group: 0.66 ± 0.26%, *p* < 0.05) (figure 3*b*). For the HE-stained paraffin sections, we compared the intima to the media thickness ratio during the formation and regression of atherosclerosis. The results showed that endothelial cells were significantly proliferated in the model group, whereas they were relatively decreased in the recovery group (control group: 0; model group: 4.43 ± 0.15; recovery group: 1.21 ± 0.09, *p* < 0.05) (figure 3*c*).

Taken together, the above results demonstrated that our rabbit atherosclerosis model was successfully established and that atherosclerosis partly regressed after feeding a caloric restriction diet in our study.

### 3.4. Differentially expressed genes were mainly enriched in four clusters

**Figure 4.**
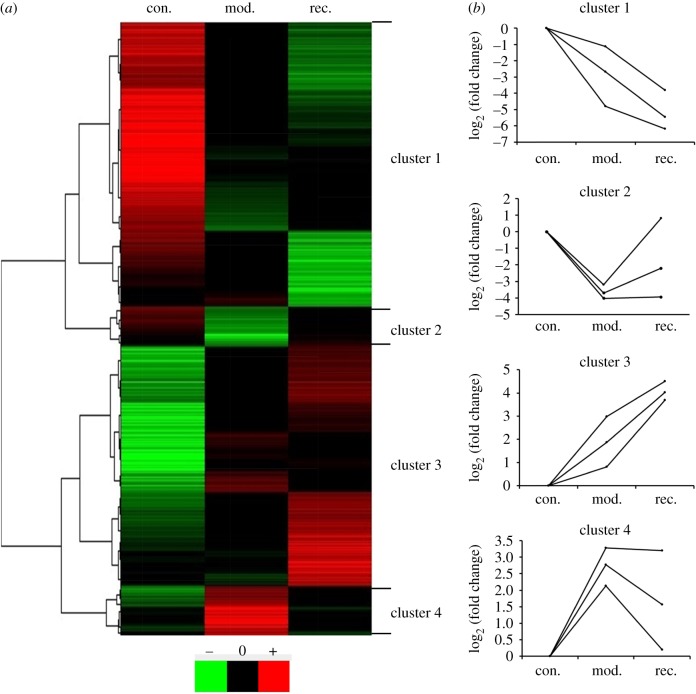
Differentially expressed genes were mainly enriched in four clusters. Cluster analysis of genes with altered expression was performed using microarray analysis, mRNA levels were analysed among control (con.), model (mod.) and recovery (rec.) groups. (*a*) The tissue screen identified 3716 genes whose mRNA levels changed at least twofold when comparing the data from any two groups. Green, black and red represent lower, normal and higher mRNA expression relative to that of control, respectively. The 3716 genes were classified into four clusters (clusters 1–4) using TreeView software. (*b*) Representative changes of dynamic gene expression curves for each cluster. Expression levels of each gene were compared with regard to the different status of rabbit atherosclerosis.

To investigate the gene changes in the progression and regression of atherosclerosis, we collected samples from the control, model and recovery groups to perform microarray analysis using gene chip analysis. We found that 3716 (37%) of 10 152 genes demonstrated statistically significant differential expression in the progression and regression of rabbit atherosclerosis (*p* < 0.05). Using TreeView software, dynamic changes in gene expression were identified at different levels of atherosclerosis (figure 4*a*). The differentially expressed genes in the progression and regression of atherosclerosis were mainly enriched in four clusters according to analysis by Cluster 3.0 software, and the representative changes in mRNA patterns were categorized from cluster 1 to 4 (figure 4*b*) (The list of genes is provided in the electronic supplementary material, table S1).

### 3.5. Gene ontology enrichment analysis of the four clusters

**Figure 5.**
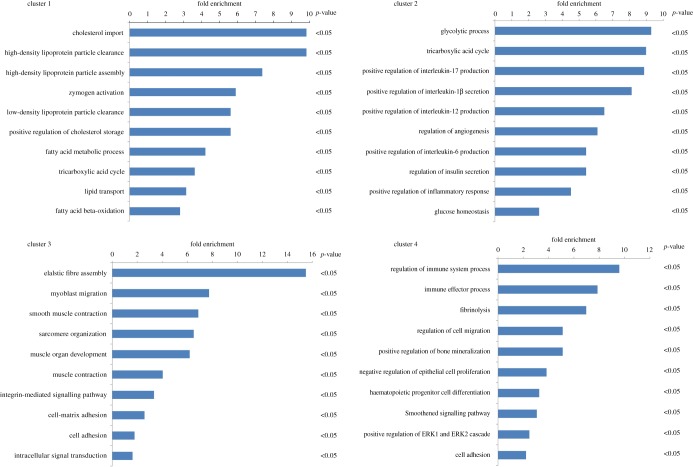
The GO enrichment analysis of the four clusters. The genes of the four clusters were studied by GO enrichment analysis using the DAVID tool. There were 1476, 629, 989, and 622 differential expression genes in clusters 1, 2, 3 and 4, respectively. A *p*-value of less than 0.05 was considered significant.

Combined with gene ontology (GO) enrichment analysis using the DAVID tool, we found that the 1476 genes in cluster 1 that initially decreased rapidly and remained reduced were enriched in the transport of cholesterol, HDL-c, LDL-c and lipid metabolism (figure 5*a*). This result suggested that continuously decreased lipid-metabolism-related genes result in excess lipids being unable to be transported out of the vascular endothelium in a timely manner. Consequently, accumulated lipids form plaques and gradually promote atherosclerosis. The 629 differentially expressed genes of cluster 2 were enriched for the inflammatory response and glucose metabolism; these genes initially decreased rapidly and then remained unchanged or returned to baseline (figure 5*b*). This finding showed that some glucose-metabolism-related genes returned to baseline after the caloric restriction diet and reduced atherosclerosis. In addition, the genes associated with the inflammatory response may be anti-inflammatory factors and were restored to normal levels after a caloric restriction diet. The 989 differentially expressed genes in cluster 3 that increased initially and continued to be upregulated in the recovery group were mainly enriched in muscle contraction, muscle structure development and muscle functions (figure 5*c*). This result indicated that hyper-proliferative smooth muscle cells partially turned into lipid-accumulated macrophage-like cells, resulting in plaque formation and arterial stenosis. There were 622 differentially expressed genes in cluster 4, mainly enriched in the regulation of the immune system process, fibrinolysis, the collagen catabolic process and cell migration, which rapidly increased and then remained unchanged or returned to baseline (figure 5*d*). This result suggested that genes related to inflammation, excessive secretion of extracellular matrix or migration may accelerate plaque formation, and they gradually returned to normal expression levels after caloric restriction diet and reduction of atherosclerosis (the genes identified in the enriched GO analysis are listed in the electronic supplementary material, table S2).

The above results indicate that the genes associated with inflammation and extracellular matrix returned to normal levels or close-to-normal levels much earlier than the genes associated with metabolism (in cluster 1) and sarcoplasmic proliferation (in cluster 3). On the other hand, the genes in cluster 2 or 4 were worthy of attention because they may play important roles in the pathological process of atherosclerosis and have the ability to more quickly regress atherosclerosis. Therefore, we preferentially screened candidate biomarkers from the genes in clusters 2 and 4.

### 3.6. Validation of the candidate biomarkers of human coronary artery disease

**Figure 6.**
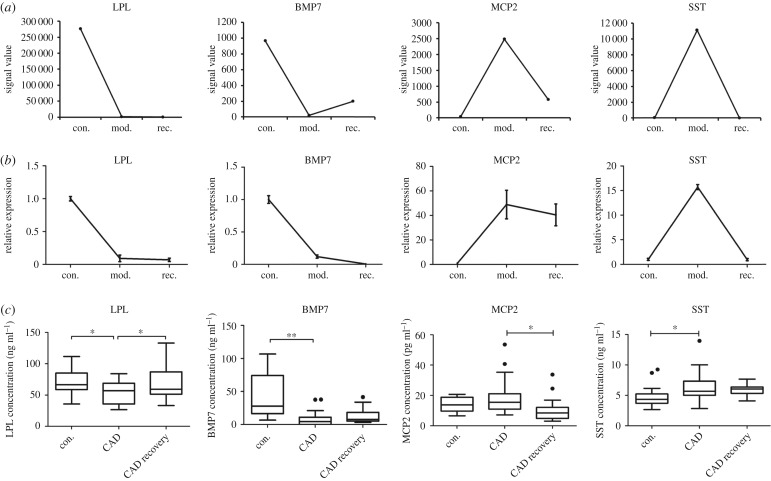
Validation of the candidate biomarkers of human coronary artery disease. (*a*) Four candidate genes were shown by signal value. (*b*) Four differentially expressed genes were selected and their mRNA levels were measured using RT-PCR (*n* = 4) and (*c*) their protein concentrations in serum were measured using ELISA (con (*n* = 16), CAD (*n* = 28), CAD recovery (*n* = 20)). Data were reported as means + s.d. or median ± IQR and among three-group comparisons were performed by ANOVA. *p < 0.05, **p < 0.01. A *p*-value of less than 0.05 was considered significant.

Under the guidance of gene chip analysis, we screened candidate biomarkers from the genes in clusters 2 and 4 because they may have the ability to more quickly regress atherosclerosis. Thus, we selected lipoprotein lipase (LPL) and bone morphogenetic protein 7 (BMP7) from the enriched genes of the ‘glycolytic process' and ‘glucose homeostasis' in cluster 2, macrophage cationic peptide 2 (MCP2) and somatostatin (SST) from the enriched genes of ‘regulation of immune system process' and ‘cell migration' in cluster 4, all of which had significantly changed signal values in the formation or regression of atherosclerosis (figure 6*a*).

First, we validated the microarray chip data using RT-PCR and found that the mRNA levels of the four candidate genes were all consistent with the microarray analysis (figure 6*b*). The results suggested that our gene chip was reliable.

Considering the four genes associated with the formation and recovery of atherosclerosis, we further investigated whether the four candidate proteins could predict the occurrence and prognosis of human CAD. We recruited healthy male subjects, patients with CAD and patients with CAD recovery. The baseline clinical characteristics of the patients are presented in table 2. Body mass index, systolic blood pressure and diastolic blood pressure were significantly higher while HDL-c concentrations were significantly lower in patients with CAD and patients with CAD recovery than in control subjects. However, age, fasting blood glucose, TCH, TG and LDL-c concentrations in the three groups were not significantly different.

**Table 2.** The baseline clinical characteristics of CAD patients. Statistical comparisons among the three groups were performed by ANOVA. Values are means ± s.d. CAD, coronary artery disease; BMI, body mass index; SBP, systolic blood pressure; DBP, diastolic blood pressure; FBG, fasting blood glucose; TCH, total cholesterol; TG, total triglyceride; LDL-c, low-density lipoprotein cholesterol; HDL-c, high-density lipoprotein cholesterol.

	controls	CAD	CAD recovery	
	(*N* = 16)	(*N* = 28)	(*N* = 20)	ANOVA
age (years)	55.13 ± 13.68	55.79 ± 10.57	59.25 ± 8.95	0.346
BMI (kg m^−2^)	21.99 ± 1.78	24.56 ± 2.49	25.17 ± 2.70	<0.001
SBP (mmHg)	117.29 ± 16.02	133.18 ± 17.19	125.90 ± 17.80	<0.001
DBP (mmHg)	73.07 ± 10.90	79.32 ± 10.08	71.4 ± 9.54	0.02
FBG (mmol l^−1^)	5.04 ± 0.53	5.96 ± 2.96	5.47 ± 1.42	0.423
TCH (mmol l^−1^)	4.31 ± 1.63	3.86 ± 1.25	4.24 ± 0.79	0.413
TG (mmol l^−1^)	1.74 ± 1.08	1.69 ± 0.77	2.32 ± 1.91	0.224
HDL-c (mmol l^−1^)	1.42 ± 0.33	1.08 ± 0.41	1.02 ± 0.23	<0.001
LDL-c (mmol l^−1^)	2.80 ± 0.19	2.34 ± 0.96	2.35 ± 0.73	0.195

We measured the concentrations of the four candidate proteins in serum by ELISA. The results are shown in figure 6*c*. As expected, the proteomic profiles of the four markers were all consistent with the mRNA levels. Serum LPL concentrations were significantly decreased in patients with CAD but significantly increased in patients with CAD recovery (control: 70.65 ± 5.94 ng ml^−1^; CAD: 54.02 ± 3.66 ng ml^−1^; CAD recovery: 69.09 ± 5.23 ng ml^−1^, *p* < 0.05). Serum BMP7 concentrations were significantly decreased in patients with CAD and showed an increased trend in patients with CAD recovery (control: 31.6 ± 8.40 pg ml^−1^; CAD: 8.78 ± 2.37 pg ml^−1^; CAD recovery: 13.42 ± 2.27 pg ml^−1^, *p* < 0.05). On the other hand, the concentrations of serum MCP2 demonstrated an increased trend in patients with CAD and significantly decreased in patients with CAD recovery (control: 13.89 ± 1.36 pg ml^−1^; CAD: 18.87 ± 1.98 pg ml^−1^; CAD recovery: 10.25 ± 1.52 pg ml^−1^, *p* < 0.05). Serum SST concentrations were significantly increased in patients with CAD and displayed a decreased trend in patients with CAD recovery (control: 4.87 ± 0.43 ng ml^−1^; CAD: 6.31 ± 0.45 ng ml^−1^; CAD recovery: 5.92 ± 0.24 ng ml^−1^, *p* < 0.05).

These findings suggest that serum LPL concentrations can be applied to simultaneously predict the occurrence and prognosis of CAD (a detailed study of the association between CAD and LPL, including other lipases, has been summarized and submitted elsewhere). In addition, serum BMP7 and SST concentrations may be indicators of CAD, and serum MCP2 concentrations may indicate the prognosis of CAD. From a technical point of view, the gene chip analysis of the progression and regression of rabbit atherosclerosis improved the efficiency of discovering indicators for the simultaneous diagnosis and prognosis of human CAD.

## 4. Discussion

In this study, we established a rabbit model of progression and regression of atherosclerosis through balloon injury followed by feeding a high-cholesterol diet or a caloric restriction diet. Then, we applied gene chip analysis to explore the expression changes of genes in the progression and regression of atherosclerosis. We found that the differentially expressed genes were mainly enriched in four clusters, and genes associated with inflammation and extracellular matrix were returned to normal or close-to-normal levels much earlier than genes associated with metabolism and sarcoplasmic proliferation, which were maintained as downregulated or upregulated for a long time after feeding a normal diet. Furthermore, candidate regulators were identified, which could be used as novel biomarkers and targets for improving the diagnosis and prognosis of human CAD in the future.

We selected rabbits as an animal model of atherosclerosis because they are very sensitive to a high-cholesterol diet, and their lipid metabolism is similar to humans [19]. By contrast, wild-type mice are highly resistant to atherosclerotic development because they have more HDL than LDL [20]. Therefore, genetically modified mice, such as apoE^−/−^ and LDLR^−/−^ mice, are more commonly used for studying atherosclerosis because they can more easily develop atherosclerosis even when fed a normal chow diet [21,22]. However, considering the deficiency of apoE genes, apoE^−/−^ mice are unlikely to self-heal upon feeding a caloric restriction diet and do not exhibit the regression of atherosclerosis. Therefore, a rabbit model is more suitable for simulating the progression and regression of atherosclerosis in humans.

In our previous research, we found that the levels of TCH and LDL-c were also reduced by feeding a normal diet ad libitum (electronic supplementary material, figure S1). The reductions were not significant after feeding a normal diet for four weeks but became significant after feeding a normal diet for eight weeks. The results of IVUS detection also showed that the lesion did not significantly reduce after feeding a normal diet for only four weeks (electronic supplementary material, figure S2). These results suggested that a normal diet can induce the regression of atherosclerosis but may require eight or more weeks. To speed up the process, we planned to regress atherosclerosis rapidly by a caloric restriction diet. Combined with our recent results, we found that caloric restriction accelerated the regression of atherosclerosis. However, as the recovery period in this study was only one month, atherosclerosis plaques did not completely regress, but at least stopped growing. This result is consistent with a previous report [5]. Importantly, the duration of both atherosclerosis progression and regression modelling in our study was shorter than that in previous reports, which was likely due to the use of a 1% cholesterol diet and a caloric restriction diet in the recovery period. This result indicates that our rabbit model is very valuable for simultaneously studying the progression and regression of atherosclerosis. Additionally, to reduce the risk of death of rabbits by femoral artery cutdown, we used the uninjured control instead of the sham control [14]. It is well known that balloon injury causes intima hyperplasia, which is widely used in the rabbit model of atherosclerosis. The gene changes between the model group and the sham control group may highlight the genes associated with the arterial wall that were injured by balloon from the diaphragm to the iliofemoral artery. Thus, we suggest that femoral artery cutdown in a sham control would not influence the gene changes in atherosclerosis.

The gene microarray technique can be used to simultaneously evaluate genome-wide changes in gene expression, thus providing a better understanding of the pathogenesis of disease. We compared the gene expression of the progression and regression of atherosclerosis by gene chip analysis. Based on enrichment analysis, we found that atherosclerosis was a pathological process closely related to lipid metabolism and inflammation [23–25]. Most genes associated with lipid metabolism, such as cholesterol import, high-density particle clearance and assembly, low-density lipoprotein particle clearance, lipid transport and so on, were downregulated after feeding the caloric restriction diet. In addition, the majority of genes associated with sarcoplasmic proliferation, such as elastic fibre assembly, myoblast migration and smooth muscle contraction, remained upregulated after feeding the caloric restriction diet, which is consistent with the fact that atherosclerosis in our rabbit model was partly regressed. On the other hand, our results suggested that these genes require more than one month to be restored during the regression of atherosclerosis.

Interestingly, we also found that genes associated with inflammation and extracellular matrix were returned to normal or close-to-normal levels much earlier. These results suggest that inflammation plays a major role in atherosclerosis [23,26]. Atherosclerotic plaque formation appears to be mediated by a chronic inflammatory process resulting from interactions among macrophages, oxidized cholesterol and cellular components of the arterial wall [26]. Key components of this process are the proliferation and migration of smooth muscle cells and the deposition of extracellular matrix [11]. This proliferative effect appears to be mediated by high levels of insulin and other growth factors, including platelet-derived growth factor [27]. On the other hand, atherosclerotic plaque regression may be mediated by enhancement of reverse lipid transport removing cellular but also extracellular lipid deposits, which may be regulated by increased insulin resistance, oxidative stress resistance and reduced inflammation. Thus, LPL and BMP7, which are associated with insulin and glucose homeostasis, and MCP2 and SST, which are associated with regulation of immune system processes and cell migration, may play important roles in both the formation and regression of atherosclerosis. We tested and verified the candidate genes by RT-PCR and ELISA and found that gene expression was consistent with the chip results. Expectedly, the correlation analysis performed using clinical data revealed that the expression levels of candidate genes were closely correlated with the occurrence and prognosis of human CAD, indicating their potential use as CAD biomarkers.

LPL, as a triglyceride lipase, plays a central role in lipid metabolism. However, it may pose pro-atherogenic or anti-atherogenic effects depending on its location [28]. In our study, the mRNA levels of LPL were downregulated by feeding a high-cholesterol diet but remained unchanged after feeding a caloric restriction diet. These results indicated that the expression of LPL was seriously suppressed by feeding a high-cholesterol diet and restored to normal levels after feeding a caloric restriction diet; however, one month may not be sufficient to restore LPL in a timely manner. The serum concentrations of LPL in CAD patients were significantly decreased, which was consistent with a previous study [29]. Moreover, serum LPL concentrations returned to normal levels in CAD patients during the recovery period. This finding suggested that LPL was closely correlated with the progression and regression of atherosclerosis and might be a good indicator of the diagnosis and prognosis of CAD.

BMP7, a member of the TGF-β superfamily, is well known for its osteogenic properties [30,31]. However, a new role of BMP7 in brown adipogenesis and energy expenditure has been reported in recent years [32,33]. In this study, we found that the mRNA levels of BMP7 were significantly decreased by feeding a high-cholesterol diet but exhibited an increasing trend after feeding a caloric restriction diet. This finding suggests that BMP7 is correlated with energy expenditure. As expected, we found that serum BMP7 concentrations were significantly decreased in patients with CAD, which suggests that BMP7 is important in the occurrence of CAD and could be used as a biomarker.

MCP2, also known as CCL8 in humans, belongs to the CC chemokine subfamily [34], displays chemotactic activity for monocytes, lymphocytes and basophils and is also involved in atherosclerosis [26]. In this study, we found that the mRNA levels of MCP2 were significantly increased by feeding a high-cholesterol diet and returned to normal levels after feeding a caloric restriction diet. In addition, serum MCP2 concentrations were significantly decreased in patients with CAD recovery. These results suggest that MCP2 may play an important role in the regression of atherosclerosis and may be a good indicator of the prognosis of CAD.

SST regulates multiple biological functions by acting through a family of five G protein-coupled receptors, somatostatin receptors (SSTRs) 1–5. SST can inhibit the activation of Rho, the assembly of focal adhesions and actin stress fibres, and cell migration through SSTR1 [35]. Our microarray and RT-PCR results indicated that the mRNA levels of SST were increased by feeding a high-cholesterol diet and began to be restored to baseline levels after feeding a caloric restriction diet. In addition, serum SST concentration was also significantly increased in patients with CAD; however, this result was not consistent with that of a previous study in which plasma SST levels were lower in hyperlipidaemic subjects than in normolipidaemic subjects [36]. This finding may be explained by the fact that the serum SST concentrations in our study were correlated with cell migration rather than insulin resistance.

Given that vascular tissue is not readily accessible in humans, identifying protein markers in the serum can have practical implications in developing diagnostic tools for the diagnosis of CAD. However, there may not be a direct correlation between vascular gene expression and serum protein levels for the same markers because of the lack of vascular tissue. In our study, we used a rabbit model to validate the correlation between vascular gene expression and serum protein levels for the same markers. We found that gene expression in serum was consistent with the chip results. Although we correlated rabbit vascular gene expression with serum protein levels in humans, the results are meaningful due to the molecular similarities between experimental rabbit atherosclerosis and human atherosclerosis. Thus, it is possible that the rabbit atherosclerosis model could be used to study the molecular pathophysiology of atherosclerosis. We believe that these findings highlight a new method that could be used to discover novel biomarkers for human CAD.

In summary, using the rabbit genome-wide chip to select differentially expressed genes in the progression and regression of rabbit atherosclerosis is an efficient and reliable method to explore important genes related to the diagnosis and prognosis of human CAD. Newly identified genes could be used as novel biomarkers and drug targets for CAD in the future.

## Supplementary Material

supplementary_figures

## Supplementary Material

Supplementary Table 1

## Supplementary Material

Supplementary Table 2

## Data accessibility

Raw sequence data have been deposited in the Gene Expression Omnibus (GEO) database of NCBI with the accession number: GSE122669.
