# Supplementary material for: Large-scale gene analysis of rabbit atherosclerosis to discover new biomarkers for coronary artery disease
Source: Open Biol. 2019 Jan 23;9(1):180238. doi: 10.1098/rsob.180238 (PMC6367139; doi:10.1098/rsob.180238)
Supplement: supplementary_figures [file rsob180238supp1.docx]

**Large-scale gene analysis using a rabbit atherosclerosis model to discover new biomarkers for coronary artery disease**

Xiaolan Yu^1^, Wen Guan^3^, Yang Zhang^1^, Qing Deng^1^, Jingjing Li^2^, Hao Ye^2^, Shaorong Deng^2^, Wei Han^2*^, Yan Yu^1*^


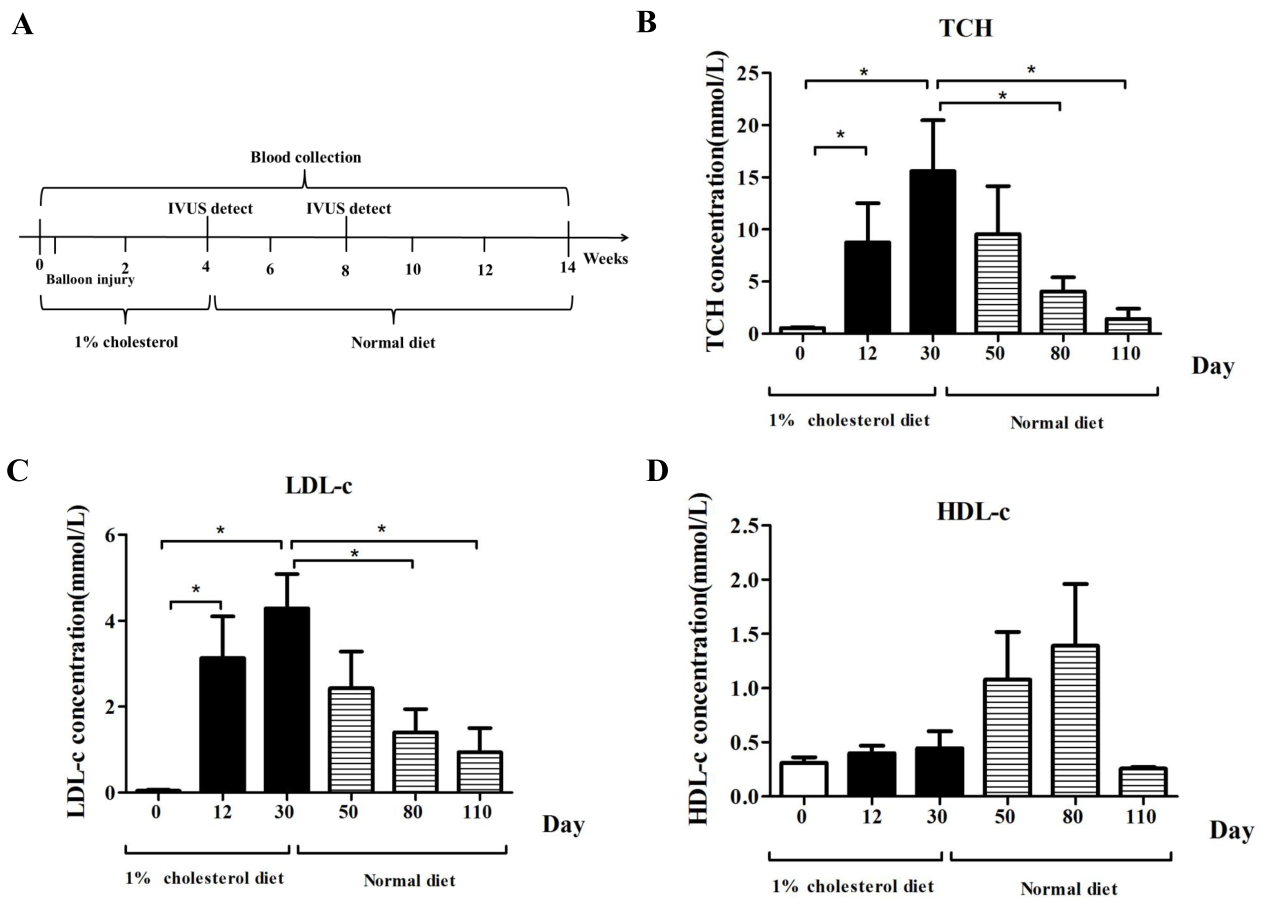


**Supplementary Figure 1.** Lipid profiles of rabbits with atherosclerosis. A schematic diagram of the development of the rabbit atherosclerosis model is shown in Figure A. Rabbits were fed a high-cholesterol diet (purified rabbit chow supplemented with 1% cholesterol) for four weeks. Three days into the diet, balloon injury of the abdominal aorta was performed. Once lesions formed, the rabbits were switched to a normal diet (chow with no added cholesterol or fat) for regression. Blood was collected every two weeks for lipid measurement. Intravascular ultrasound imaging was performed at the end of the 4th and 8th weeks to detect the characteristic lesions. TCH, LDL-c and HDL-c concentrations are presented in B, C, and D, respectively. Data were reported as the means ± SD, and among three-group comparisons were performed by anova, n=4. A *P*-value of less than 0.05 was considered significant.

**
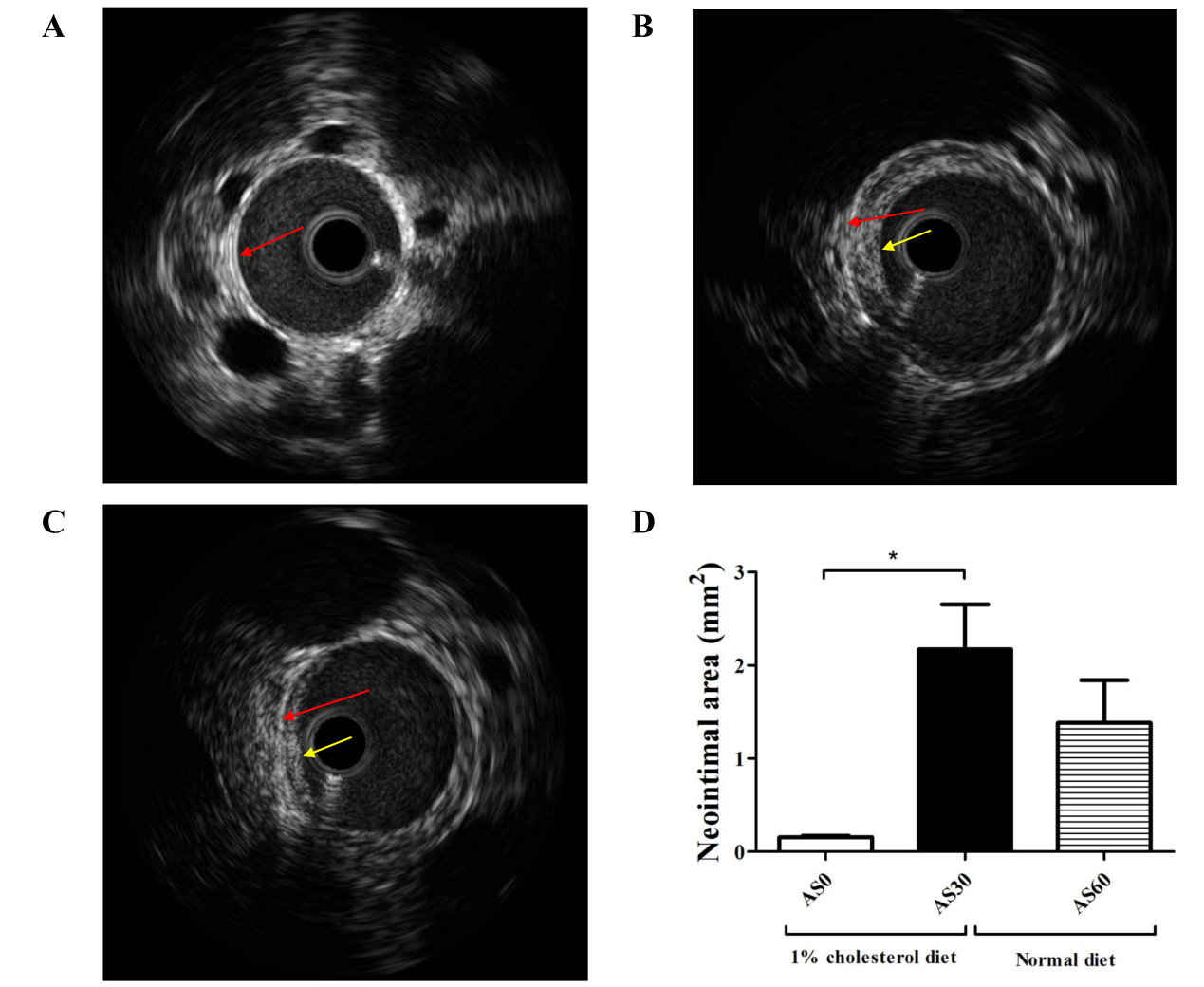
**

**Supplementary Figure 2.** Intravascular ultrasound of atherosclerosis in the rabbits. Intravascular ultrasound images show representative pictures of the abdominal aorta in control rabbits (A) and during the formation (B) and regression (C) of atherosclerosis (AS). Obvious neointima was observed on the 30th day of AS, and some neointima was observed on the 60th day of AS, whereas no neointima was observed at AS0. The mean neointimal area was measured and analysed (D). The red and yellow arrows indicate the intima and the neointima, respectively. Data were reported as the means ± SD, and among three-group comparisons were performed by anova, n=4. A *P*-value of less than 0.05 was considered significant.
